# Effect of Autologous Bioactive Concentrated Growth Factor and Residual Dental Pulp on Dentin–Pulp Complex Regeneration

**DOI:** 10.3390/biomedicines14030537

**Published:** 2026-02-27

**Authors:** Abeer Ezat Wahba, Safwat Elwaseef, Huda Ibrahim Mostafa, Weal B. Abdelhameed, Ahmed Mostafa Abbas, Ashraf Mohamad Emran

**Affiliations:** 1 Department of Oral and Dental Biology, Faculty of Dental Medicine for Girls, Al-Azhar University, Cairo 11884, Egypt; 2 Department of Endodontics, Faculty of Dental Medicine, Al-Azhar University, Asuit 71524, Egypt; 3 AEGD, Eastman Institute for Oral Health, University of Rochester, Rochester, NY 14620, USA; 4 Department of Endodontics, Faculty of Dental Medicine for Girls, Al-Azhar University, Cairo 11884, Egypt; drhudaabdo2010@azhar.edu.eg; 5 Department of Oral Biology, Faculty of Dental Medicine, Al-Azhar University, Asuit 71524, Egypt; 6 Department of Oral Biology, Faculty of Dental Medicine, Al-Azhar University, Cairo 11884, Egypt

**Keywords:** regeneration, autologous bioactive concentrated growth factor, residual dental pulp

## Abstract

**Background/Objectives:** This study aimed to evaluate the regenerative potential of autologous concentrated growth factor (CGF) combined with residual pulp tissue in immature dog teeth using a histological and histomorphometric analysis. **Materials and Methods:** Thirty immature anterior and premolar teeth, harvested from four dogs, were randomly assigned to three groups (*n* = 10 each): group I (negative control, untreated teeth), group II (positive control, complete pulp removal with blood clot in the canal), and group III (experimental, partial pulp removal with 1–4 mm residual pulp and placement of autologous CGF). After 1 and 3 months, animals were euthanized, and samples were processed for histological and histomorphometric assessments. **Results**: The CGF-treated group exhibited newly formed tissue with morphological characteristics comparable to the negative control group after partial pulp removal. **Conclusions**: The combination of CGF with 1–4 mm of residual pulp was associated with enhanced tissue organization, representing a promising approach for dentin–pulp complex (DPC) regeneration within this experimental context.

## 1. Introduction

DPC, derived from the dental papilla, contains a diverse population of cells supported by vascular and neural networks. It governs dentin formation, nutrition, defense, and repair, making it indispensable for tooth vitality. Because it is encased within rigid dentin and lacks collateral circulation, the pulp has little tolerance for inflammation. Consequently, insults such as caries, trauma, or microbial invasion can quickly overwhelm its defenses, leading to pulpitis or necrosis and often necessitating endodontic treatment. Recent advances in regenerative endodontics, however, have challenged the traditional belief that exposed pulp is irreversibly damaged, highlighting its intrinsic healing potential and the possibility of biologically based therapies to restore function [1,2].

Once inflammation occurs, the accumulation of toxic byproducts within the pulp chamber raises intrapulpal pressure and compromises microcirculation, driving progression to irreversible pulpitis and extension to periapical tissues. At this stage, root canal treatment is required. The procedure involves removal of the pulp, followed by mechanical debridement, chemical disinfection, and obturation of the canal space with inert materials. Historically, apexification with calcium hydroxide or mineral trioxide aggregate (MTA) has been used, but these approaches induce apical closure without promoting further root maturation. While root canal treatment effectively eradicates infection and preserves the tooth, the loss of pulp vitality leaves it devitalized, brittle, and discolored, with increased risk of fracture. Preserving pulp vitality is therefore clinically important, as it supports continued root development, maintains natural biomechanics, and improves long-term survival [3,4].

In immature permanent teeth, conventional root canal treatment not only presents technical difficulties due to wide canals, thin dentinal walls, and open foramina but also eliminates the biological stimulus for continued root development. This clinical dilemma underscores the need for biologically based approaches capable of preserving or re-establishing pulp vitality while promoting root maturation [5,6,7].

Regenerative endodontic therapy (RET) has emerged as a biologically based alternative, drawing on the principles of tissue engineering to restore a functional DPC. RET strategies can broadly be categorized into cell homing, which stimulates the migration of endogenous stem or progenitor cells into the canal space; cell transplantation, which delivers exogenous stem cells directly into the pulp chamber. Successful outcomes are largely attributed to stem cells from the apical papilla and dental pulp stem cells (DPSCs), which provide the cellular foundation for dentin–pulp regeneration. In both approaches, a suitable scaffold is critical for supporting cell adhesion, proliferation, and differentiation. Natural scaffolds such as a blood clot, platelet-rich plasma (PRP), platelet-rich fibrin (PRF), and CGF not only provide a three-dimensional matrix but also release bioactive molecules that guide tissue regeneration. Together, these elements create the biological environment necessary for pulp regeneration and continued root maturation [8,9,10].

CGF, first described by Sacco in 2006, is an autologous platelet concentrate obtained by variable-speed centrifugation of venous blood without anticoagulants. Compared with PRP and PRF, CGF yields a more fibrin scaffold capable of entrapping higher concentrations of bioactive molecules [Vascular Endothelial Growth Factor (VEGF) and Transforming Growth Factor-beta 1 (TGF-β1)] and progenitor cells. These bioactive molecules are released more gradually, stimulating cellular proliferation and supporting predictable tissue repair [11,12,13,14,15]. The dense fibrin matrix of CGF allows for a sustained release of these molecules, which is critical for the recruitment of DPSCs and the promotion of angiogenesis [16,17]. By preserving 1–4 mm of residual pulp, this study utilizes these tissues as ‘biological signaling centers’, providing the necessary neurovascular cues and native stem cell niches required to guide the differentiation of odontoblast-like cells [18]. The enhanced fibrin strength also improves handling during surgical use, broadening its applicability in RET, periodontal therapy, and implant-related procedures [19,20,21]. CGF is established as a rich source of growth factors in surgical healing; however, its hypothetical benefits in facilitating specific odontoblastic differentiation within the pulp chamber require further preclinical validation.

The most regenerative endodontic strategies rely solely on cell homing from apical tissues using blood clot or platelet concentrates, while the biological contribution of deliberately preserved residual pulp tissue remains insufficiently investigated. The present study lies in the intentional combination of autologous CGF with a standardized amount of residual vital pulp (1–4 mm), aiming to exploit both the scaffold-driven regenerative potential of CGF and the intrinsic biological signaling capacity of remaining pulp tissue. Moreover, this study aimed to characterize the histological and histomorphometric features of tissue formed after the application of autologous CGF combined with residual pulp. The null hypothesis of the present study was that the application of autologous CGF combined with residual pulp tissue would not result in significantly different histological or histomorphometric regenerative outcomes in immature dog teeth compared with the control treatment.

## 2. Materials and Methods

### 2.1. Animal Housing

For this investigation, four healthy dogs (6–8 months old) of equal sex distribution were selected. The animals were acclimatized for two weeks and confirmed to be clinically normal prior to the experiment. They were housed individually in cages at the Department of Veterinary Surgery, Veterinary Hospital, El Abbasia, under conditions of adequate ventilation, controlled humidity, and a soft-food diet. Every effort was made to minimize both the number of animals used and their suffering. All experimental procedures were conducted in accordance with ethical standards and approved by the Research Ethics Committee (REC) of the Faculty of Dental Medicine for Girls, Al-Azhar University (Code: REC-PD-25-27).

### 2.2. Sample Selection and Grouping

Thirty immature anterior and premolar teeth were included. From each dog, teeth were randomly assigned to 3 groups (*n* = 10 per group):

- Group I (Control): Teeth left intact with no intervention.
- Group II (Blood Clot): Pulp tissue is completely removed with sterile stainless steel endodontic broaches, and canals are allowed to fill with blood clots.
- Group III (CGF): Pulp tissue removed, canals enlarged with large K-files (100–120 file sizes) to 1–4 mm short of the apex, then filled with CGF.

### 2.3. Sample Size Calculation


$$f=\frac{\sigma_{\mu }}{\sigma }$$

$$\sigma_{\mu }^{2}=\frac{\sum_{i=1}^{k}n_{j}{\left(\mu_{i}-\mu \right)}^{2}}{N}$$

Sample size was calculated using G*Power software, version 3.1.9.2 (University of Kiel, Kiel, Germany) [22]. Parameters included an effect size (f) of 0.76 (large), α = 0.05, β = 0.05, and power (1 − β) = 0.95, based on mean differences reported in previous studies [23]. The estimated sample size was 30 teeth, which were divided equally among the 3 experimental groups as described above.

### 2.4. Dog Anesthesia

All procedures were performed under aseptic conditions with sterile instruments. Dogs were fasted for 12 h before general anesthesia. Premedication was administered 15 min preoperatively with xylazine HCl (1 mg/kg, IM) and atropine sulfate (0.1 mg/kg, SC). Anesthesia was induced with ketamine HCl (5 mg/kg, IV via the cephalic vein). Anesthesia was maintained with thiopental sodium administered intravenously (2.5 mg/kg), with supplemental boluses of 3 mg/kg as required [23].

### 2.5. Preparation of CGF

Venous blood (10 mL) was collected from each dog into sterile disposable non-anticoagulant tubes. Samples were processed in a Medifuge centrifuge (Sil Fradent Srl, Sofia, Italy) using the manufacturer’s one-step program: acceleration 30 s; 2700 rpm for 2 min; 2400 rpm for 4 min; 2700 rpm for 4 min; 3000 rpm for 3 min; deceleration 36 s, then stop. Three layers formed: an upper serum phase; a middle fibrin buffy coat (CGF layer) containing aggregated platelets and growth factors; and a lower red blood cell (RBC) phase. The CGF layer was separated from the RBC phase with microsurgical scissors, retaining a minimal amount of RBCs. The CGF membrane was then compressed between sterile gauze to express excess fluid and trimmed into small pieces [24].

### 2.6. Procedures

All endodontic procedures were performed by two experienced endodontists under standardized aseptic conditions to minimize operator-related variability. Incomplete root formation was confirmed radiographically for all teeth. Experimental teeth (Groups II and III) received endodontic access cavities prepared with a #2 round carbide bur followed by a stone bur in a low-speed handpiece; teeth and burs were irrigated with sterile normal saline to prevent heat-related damage during preparation. After pulpectomy, no root irrigation was performed [18].

- Group II (Blood Clot): Pulp tissue was completely extirpated with sterile barbed broaches. An intracanal blood clot formed from periapical tissues without deliberate over-instrumentation beyond the apex.
- Group III (CGF): The amount of residual pulp tissue (1–4 mm) was standardized by controlled mechanical removal using large K-files up to sizes 100–120 under radiographic guidance. Canal enlargement was performed to a predetermined working length measured from the radiographic apex, ensuring that instrumentation stopped consistently 1–4 mm short of the apex in all Group III specimens. The same experienced operator performed all procedures using identical instruments and protocols to minimize operator-related variability. CGF prepared as described was inserted into the canals.

MTA was mixed according to the manufacturer’s instructions and placed as an orifice plug in groups II and III. The remaining access cavity was sealed with glass ionomer. After MTA placement, a sterile saline-moistened cotton pellet was placed over the material to provide the moisture required for proper setting, and the access cavity was temporarily sealed before final restoration.

### 2.7. Sample Collection

At the predetermined intervals of one and three months, the animals were euthanized through a lethal dose of pentobarbital sodium in combination with phenytoin sodium [23]. After euthanasia, the jaws were carefully excised and processed for subsequent light microscopic evaluation.

### 2.8. Histological Examination

For tissue fixation, the maxilla and mandible were extracted, dissected, and immersed in 10% buffered formalin for at least 48 h. Decalcification was subsequently carried out in a sodium citrate–formic acid solution for a period of six months. After decalcification, the specimens were dehydrated through a graded ethanol series (70% to 100%). Ultimately, xylene, which is miscible with alcohol, was used to replace the alcohol after 100% alcohol had been used to replace the water. Individual teeth, along with their surrounding alveolar bone, were carefully separated using a lancet to produce discrete tissue blocks. Longitudinal buccolingual sections were prepared at a thickness of 4–6 µm. These sections were stained with hematoxylin and eosin (H&E) as well as Masson’s trichrome for histological assessment [23]. For histomorphometric analysis, the percentage (%) of collagen-positive area was calculated using ImageJ software (ImageJ version 1.54 j, National Institutes of Health, Bethesda, MD, USA). Each specimen was examined in five non-overlapping fields. The histological and histomorphometric evaluations were performed by an experienced examiner who was blinded to group allocation, thereby reducing observational bias. Histomorphometric assessment of the percentage of collagen-positive area was performed to provide a descriptive measure of structural tissue density and should not be interpreted as a measure of functional pulp recovery.

### 2.9. Statistical Analysis

The following statistical tests were used to gather, calculate, tabulate, and statistically evaluate all of the data. The normal distribution of the samples was examined using a normality test (Shapiro–Wilk). Mean ± Standard deviation (SD) was used to calculate descriptive statistics. Time intervals were compared using paired t-tests. Group comparisons were done using one-way ANOVAs. For pairwise comparison, Tukey’s post hoc test was utilized. *p*-value ≤ 0.05 is regarded as statistically significant. The computer program SPSS software for Windows version 26.0 was used to perform all statistical analyses (Statistical Package for Social Science, Armonk, NY, USA: IBM Corp) at *p*-Value < 0.05. Because multiple teeth were obtained from each of the four animals, the potential for intra-animal correlation (clustering) exists. While statistical analysis treated each tooth as an independent observation (*n* = 30), this limitation should be considered when interpreting the data’s generalizability.

## 3. Results

### 3.1. Hematoxylin and Eosin (H&E)

#### 3.1.1. At One Month

**Figure 1:**
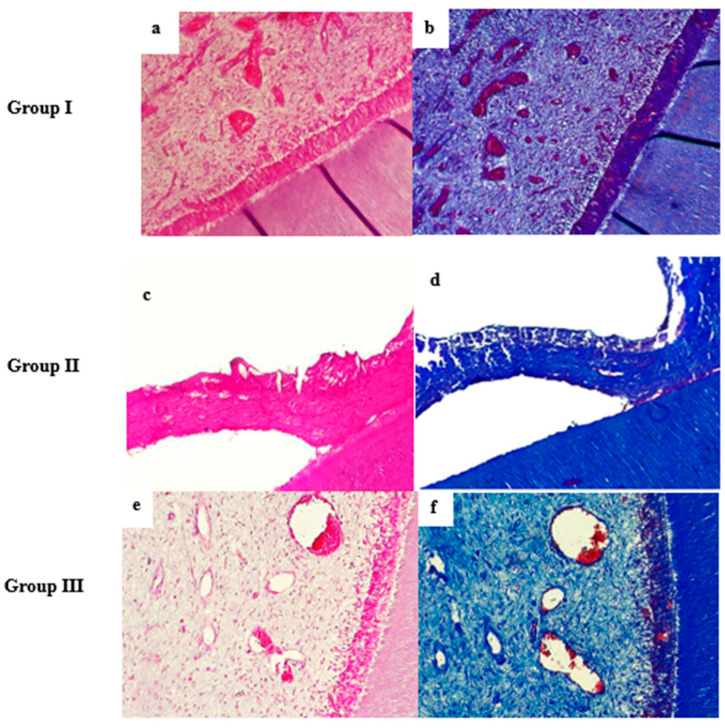
Photomicrographs of the root canals at one month: (**a**,**b**) Group I showed a normal dentin pulp structure containing abundant blood vessels, fibroblasts, collagen fibers, and a palisade-like odontoblastic cell layer. (**c**,**d**) Group II showed deformed connective tissue without blood vessels or odontoblast-like cells. (**e**,**f**) Group III showed numerous dilated blood vessels and less polarized odontoblast-like cells. (**a**,**c**,**e**) Hematoxylin–eosin staining (H&E X 200). (**b**,**d**,**f**) Masson’s trichrome staining (MTC X 200).

Histological findings from 30 teeth across four animals revealed distinct patterns. In group I, the root canals of naturally developing teeth showed a closely related tissue complex that was made up of dentin and pulp. Dentin’s structure was normal, consistent, and had somewhat curved tubules. The pulp core was filled with normal, uniform, and loose connective tissue that was composed of abundant and scattered blood vessels. The predominant pulpal cells were fibroblasts. In an amorphous ground substance, collagen fibers were found. At the dentin–pulp interface, there was a well-arranged odontoblastic layer. The nuclei of the columnar polarized odontoblasts were found at their base, and their processes extended to the dentin. They formed a palisade-like arrangement (Figure 1a).

Group II showed root canals that were filled with some deformed, disordered, and more irregular connective tissue. Moreover, it did not show the regular histologic structure of new blood capillaries, odontoblast-like cells, or dentin-like tissues along the intracanal walls (Figure 1c).

Group III showed pulp-like tissue containing abundant newly formed fibroblasts and blood vessels. Although, some of these blood vessels appeared large, dilated, and congested with blood. Distinctive odontoblast-like cells were observed; however, they were less polarized than group I (Figure 1e).

#### 3.1.2. At Three Months

**Figure 2:**
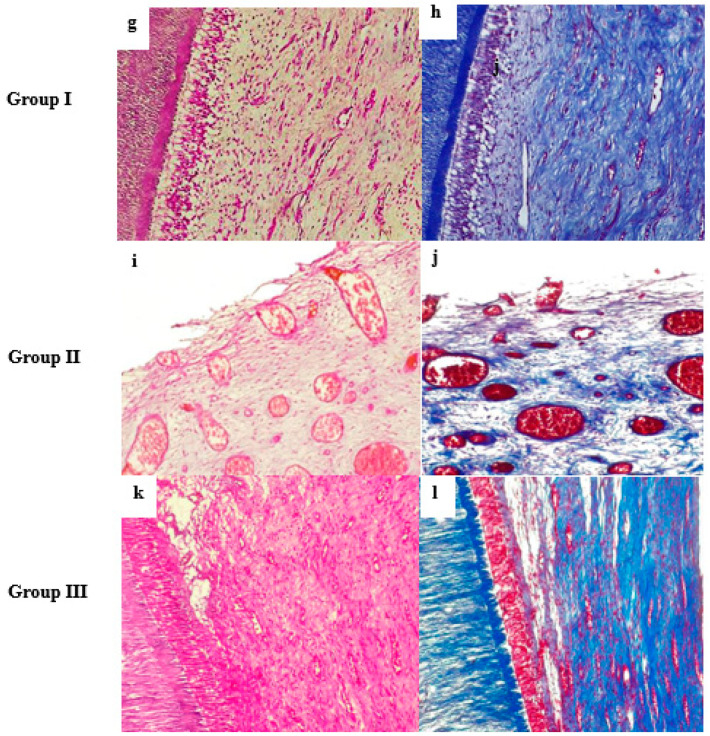
Photomicrographs of the root canals at three months: (**g**,**h**) Group I showed numerous fibroblasts, collagen fibers, blood vessels, a polarized odontoblastic cell layer, and a thick predentin layer. (**i**,**j**) Group II showed regenerated pulp-like tissue without odontoblast-like cells and containing large, dilated blood vessels congested with blood. (**k**,**l**) Group III showed well-organized pulp-like tissue, a well-formed predentin layer, and odontoblast-like cells arranged in a palisading-like manner. (**g**,**i**,**k**) Hematoxylin–eosin staining (H&E × 200). (**h**,**j**,**l**) Masson’s trichrome staining (MTC × 200).

It is interesting to note that group I at three months had the same characteristics as at one month, but they were more obvious at that time. Group I displayed abundant and more mature extracellular matrix, fibroblasts, and blood vessels. A thick layer of predentin was detected. Moreover, odontoblast-like cells were longer, more polarized, and had a more palisading arrangement than at one month (Figure 2g).

Group II showed newly formed connective tissue that was more irregular, loosely arranged, and separated from the dentinal walls. In addition, it showed newly formed fibroblasts, markedly large, dilated blood vessels, RBCs stagnation, and congestion. Moreover, odontoblast-like cells were not observed (Figure 2i).

Group III showed densely packed tissue that resembles group I in morphology. It exhibited numerous blood vessels, fibroblasts, and extracellular matrix. Palisading-arranged odontoblast-like cells and a thick predentin layer were noticed. Odontoblasts extended their processes tightly to dentinal tubules without gaps (Figure 2k).

### 3.2. Masson’s Trichrome Special Stain

At one month, group I showed uniform and mature collagen fibers (Figure 1b). Group II displayed less structured collagen fibers with no orientation (Figure 1d). Group III, however, revealed collagen fibers that were more densely arranged than those of group II (Figure 1f). At 3 months, group I showed a very organized network of dense and mature collagen fibers arrangement (Figure 2h). Group II presented more collagen fibers deposition than at one month. Although these collagen fibers remain less mature and less organized than group III (Figure 2j). Nevertheless, group III exhibited obvious pulp-like tissue with highly ordered dense collagen fibers, which appeared similar to the collagen fibers morphology in group I (Figure 2l).

Histomorphometric analysis, Table 1, showed that there were inter-group differences at 1 month and 3 months with significant differences using the ANOVA test at *p* < 0.05. For both time points, the highest mean value was recorded in group I, followed by group III, while group II had the lowest values in both times. The pairwise comparisons showed a significant difference between each group and the others, except for group I with group III at 3 months.

**Figure 3:**
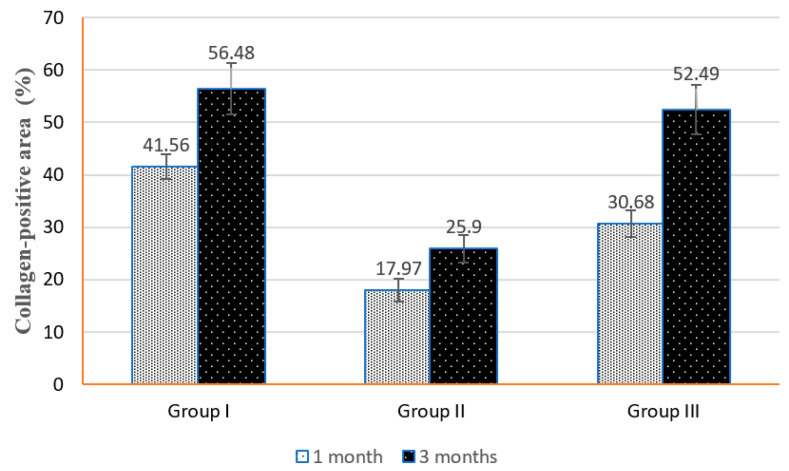
Bar charts show the comparison groups for collagen-positive area (%) at the different times.

For intra-group changes from 1 month to 3 months, all groups I, II and III exhibit significant increases in collagen-positive area percentages (group I: 41.56 to 56.48; group II: 17.97 to 25.90; Group III: 30.68 to 52.49), using paired *t* tests (*p* < 0.001 for all groups) indicating that both time progression and group assignment influence collagen fiber percentages. Overall, results indicate meaningful intra-group improvement over time and robust inter-group differences, suggesting differential responses among the groups (Figure 3).

## 4. Discussion

Based on the statistically significant histological and histomorphometric differences observed between the CGF-treated group and the control groups, the null hypothesis was rejected. The findings indicate that the combination of CGF with residual pulp tissue positively influences DPC regeneration compared with blood clot regeneration or no treatment.

Proposing that residual pulp acts as a native reservoir of DPSCs, neurovascular elements, and endogenous growth factors, while CGF provides a mechanically stable fibrin scaffold with sustained release of bioactive molecules (e.g., PDGF, VEGF, TGF-β). The interaction between these two components likely enhances angiogenesis, odontoblastic differentiation, collagen maturation, and organized pulp-like tissue formation [11,12,13,14,15]. These results support the notion that pulp preservation of surviving tissue will contribute to the enhanced scaffold-mediated regeneration, in concomitant positive association, rather than depending only on cell homing from apical tissues [8].

The promising regenerative results in the CGF group could be due to both structural stability and biological richness, offered by CGF, which has many leukocytes, platelets, and multiple growth factors released over time. CGF has a variety of benefits compared to blood clot scaffolds that are inconsistent and early degrading; CGF has mechanical stability as well as sustained release of the growth factor, promoting the morphological characteristics of odontoblastic differentiation, proliferation of fibroblasts, and angiogenesis. This is consistent with the well-aligned collagen, increased vascularity, and palisade odontoblast-like cells seen histologically in group III [3,25,26,27,28,29,30].

The remaining pulp tissue has an important biological function, serving as a native reservoir of DPSCs, neurovascular elements, and growth factors. Previous in vivo studies demonstrated that a small amount of residual healthy pulp tissue is more than able to significantly contribute to the regenerative DPC by means of identification as a biological signaling center instructing cells on differentiation and matrix formation [18,31]. The present conclusions support this explanation, as the interface between CGF and remaining pulp tissue was able to demonstrate similar features to those of the negative control group at 3 months [16,18].

The histomorphometric analysis revealed that the CGF group exhibited characteristics associated with advanced matrix maturation. The dense fibrin network potentially facilitated a stable scaffold for cell migration and collagen deposition. This suggests a synergistic effect between the sustained growth factor release from the CGF and the native signaling molecules provided by the residual pulp. While the results point toward a more predictable morphological outcome compared to blood clot induction, the lack of electrophysiological data remains a limitation for confirming total biological recovery [17,32].

On the other hand, the blood clot group displayed dilated, congested blood vessels, uneven connective tissue development, and no odontoblast-like cells, which is in line with other findings showing that blood clot-based regeneration frequently results in repair rather than real regeneration. The worst outcomes shown in this group could be explained by the blood clots’ irregular growth factor release and lack of structural stability [4,5].

Because of those similarities in anatomic, healing, and pulp biology conditions compared to human teeth, immature dog teeth were used as experimental models, which became common in the field of RET. Nevertheless, cross-species variation in immune response and healing kinetics is still a drawback [20]. Key limitations include the small number of animals (*n* = 4), the clustering of samples at the animal level, and the preclinical nature of the model, which may not fully replicate human clinical healing.

## 5. Conclusions

The clinical significance of this study lies in the shift from “repair” to “functional regeneration.” By preserving even minimal residual pulp (1–4 mm), clinicians can leverage native “biological signaling centers” to achieve better outcomes than traditional cell-homing techniques. In this preclinical descriptive study, the combination of autologous CGF with 1–4 mm of residual pulp was associated with enhanced structural tissue organization and a collagen density percentage (52.49% ± 4.70) that was statistically comparable to native pulp (56.48% ± 4.88). These findings suggest that CGF facilitates a favorable environment for the structural reconstitution of the DPC. While the presence of organized odontoblast-like cells is a promising morphological indicator, these results do not constitute proof of physiological vitality or sensory function. Future clinical trials are essential to validate the long-term functional effectiveness and clinical applicability of this approach in human subjects.

## Figures and Tables

**Table 1:** Comparison groups for collagen-positive area (%) at different times.

Group	1 Month	3 Months	Paired *t* Test	*p* Value
Group I	41.56 ± 2.40 ^a^	56.48 ± 4.88 ^a^	31.43	<0.01 **
Group II	17.97 ± 2.17 ^c^	25.90 ± 2.57 ^b^	11.58	<0.01 **
Group III	30.68 ± 2.55 ^b^	52.49 ± 4.70 ^a^	29.57	<0.01 **
ANOVA test	123.10	78.89		
*p* value	<0.01 **	<0.01 **		

** and different superscript letters (column) mean significant difference at *p* < 0.05 and 0.01.

## Data Availability

The data presented in this study are available on request from the corresponding author. The data are not publicly available due to ethical restrictions related to the use of animal experimental data.

## References

[1] Ezzat A., Adawy H.A., Abd Allah M.F. (2022). Regenerative Potentiality of Concentrated Growth Factors with Partially and Completely Amputated Pulp Tissue (Histological Study). Al-Azhar Dent. J. Girls.

[2] Taha N.A., About I., Sedgley C.M., Messer H.H. (2020). Conservative management of mature permanent teeth with carious pulp exposure. J. Endod..

[3] Yu S., Zheng Y., Guo Q., Li W., Ye L., Gao B. (2023). Mechanism of Pulp Regeneration Based on Concentrated Growth Factors Regulating Cell Differentiation. Bioengineering.

[4] Piglionico S.S., Pons C., Romieu O., Cuisinier F., Levallois B., Panayotov I.V. (2023). In vitro, ex vivo, and in vivo models for dental pulp regeneration. J. Mater. Sci. Mater. Med..

[5] Moukhtar T., Darrag A., Labib A., Ghoneim W. (2023). Revascularization Induced Maturogenesis of Non-Vital Immature Teeth Using Different Scaffolds and Intra Canal Medications. Egypt Dent. J..

[6] Mostafa H.I., Abdel Fatah D.S., Abdelkafy H. (2022). Cytotoxic Effect of Different Concentrations of Chitosan and Propolis Nanoparticles on Periodontal Ligament Stem Cells. Al-Azhar Dent. J. Girls.

[7] Li J., Zheng L., Daraqel B., Liu J., Hu Y. (2023). The efficacy of concentrated growth factor and platelet-rich fibrin as scaffolds in regenerative endodontic treatment applied to immature permanent teeth: A retrospective study. BMC Oral Health.

[8] Yan H., De Deus G., Kristoffersen I.M., Wiig E., Reseland J.E., Johnsen G.F., Silva E.J.N.L., Haugen H.J. (2023). Regenerative endodontics by cell homing: A review of recent clinical trials. J. Endod..

[9] Gandhi J.M., Gurunathan D. (2023). Regenerative Endodontic Management of An Immature Non-Vital Permanent Molar Using Concentrated Growth Factor—A Case Report. J. Popul. Ther. Clin. Pharmacol..

[10] Hargreaves K., Diogenes A., Teixeira F. (2013). Treatment Options: Biological Basis of Regenerative Endodontic Procedures. J. Endod..

[11] Sacco L. (2006). International academy of implant prosthesis and osteoconnection. Lecture.

[12] Rodella L.F., Favero G., Boninsegna R., Buffoli B., Labanca M., Scarì G., Batani T., Rezzani R. (2011). Growth factors, CD34 positive cells, and fibrin network analysis in concentrated growth factors fraction. Microsc. Res. Tech..

[13] Chen L., Cheng J., Cai Y., Zhang J., Yin X., Luan Q. (2023). Efficacy of concentrated growth factor (CGF) in the surgical treatment of oral diseases: A systematic review and meta-analysis. BMC Oral Health.

[14] Mozzati M., Gallesio G., Tumedei M., Del Fabbro M. (2020). Concentrated growth factors vs. leukocyte-and-Platelet-Rich fibrin for enhancing postextraction socket healing. a longitudinal comparative study. Appl. Sci..

[15] Bharti R., Tikku A.P., Verma P., Yadav R.K., Pant A.B. (2024). Effect of platelet-rich fibrin and concentrated growth factor on the regenerative potential of human-induced pluripotent stem cells: A comparative analysis. J. Conserv. Dent. Endod..

[16] Xie Y., Feng X., Hu Y., Wang Z., Xia X., Luo X., Xiao Y. (2023). Pulp regeneration by transplantation of dental pulp with the synergy of concentrate growth factor: An in vitro and in vivo study. Res. Sq..

[17] Bai Y., Liu X., Li J., Wang Z., Guo Q., Xiao M., Cooper P.R., Yu Q., He W. (2022). Stage-Dependent Regulation of Dental Pulp Stem Cell Odontogenic Differentiation by Transforming Growth Factor-β1. Stem Cells Int..

[18] Torabinejad M., Alexander A., Vahdati S.A., Grandhi A., Baylink D., Shabahang S. (2018). Effect of Residual Dental Pulp Tissue on Regeneration of Dentin-pulp Complex: An In Vivo Investigation. J. Endod..

[19] Firouzi N., Yavari H.R., Rahimi S., Roshangar L., Chitsazha R., Amini M. (2022). Concentrated Growth Factors Combined with Lipopolysaccharide Stimulate the In Vitro Regenerative and Osteogenic Activities of Human Dental Pulp Stem Cells by Balancing Inflammation. Int. J. Dent..

[20] Elayah S.A., Liang X., Sakran K.A., Xie L., Younis H., Alajami A., Tu J., Na S. (2022). Effect of concentrated growth factor (CGF) on postoperative sequel of completely impacted lower third molar extraction: A randomized controlled clinical study. BMC Oral Health.

[21] Li S., Yang H., Duan Q., Bao H., Li A., Li W. (2022). A comparative study of the effects of platelet-rich fibrin, concentrated growth factor and platelet-poor plasma on the healing of tooth extraction sockets in rabbits. BMC Oral Health.

[22] Faul F., Erdfelder E., Lang A.G., Buchner A. (2007). G*Power 3: A flexible statistical power analysis program for the social, behavioral, and biomedical sciences. Behav. Res. Methods.

[23] Mohamed D.A.A., Abdelwahab S.A., Mahmoud R.H., Taha R.M. (2023). Radiographic and immuno-histochemical evaluation of root perforation repair using MTA with or without platelet-rich fibrin or concentrated growth factors as an internal matrix in dog’s teeth: In vivo animal study. Clin. Oral Investig..

[24] Kavitha M., Shakthipriya S., Arunaraj D., Hemamalini R., Velayudham S., Bakthavatchalam B. (2022). Comparative evaluation of platelet-rich fibrin and concentrated growth factor as Scaffolds in Regenerative Endodontic Procedure: A Randomized Controlled Clinical Trial. J. Contemp. Dent. Pract..

[25] Li Z., Liu L., Wang L., Song D. (2021). The effects and potential applications of concentrated growth factor in dentin–pulp complex regeneration. Stem Cell Res. Ther..

[26] Divya S.B. (2023). Estimation of Release of VEGF From Concentrated Growth Factor compared with that of Platelet Rich Fibrin at Different Time Intervals. J. Biomed. Engin..

[27] Zeng Q., Zhou C., Li M., Qiu Y., Wei X., Liu H. (2023). Concentrated growth factor combined with iRoot BP Plus promotes inflamed pulp repair: An in vitro and in vivo study. BMC Oral Health.

[28] Chen L., Huang C., Zhong Y., Chen Y., Zhang H., Zheng Z., Jiang Z., Wei X., Peng Y., Huang L. (2022). Multifunctional sponge scaffold loaded with concentrated growth factors for promoting wound healing. iScience.

[29] Ruan Q., Tan S., Guo L., Ma D., Wen J. (2023). Prevascularization techniques for dental pulp regeneration: Potential cell sources, intercellular communication and construction strategies. Front. Bioeng. Biotechol..

[30] Naji M.A., Nagah R.M., Abdel-Rahman F.H., Marzook H.A. (2022). Comparison between the Effect of Bisphosphonates and Concentrated Growth Factors on Bone Healing Experimental Study. Mans. J. Dent..

[31] Astudillo-Ortiz E., Babo P.S., Sunde P.T., Galler K.M., Gomez-Florit M., Gomes M.E. (2023). Endodontic tissue regeneration: A review for tissue engineers and dentists. Tissue Eng. Part B Rev..

[32] Widbiller M., Buchalla W. (2021). Histological and molecular characterization of regenerated pulp tissue. Arch. Oral Biol..

